# Forage plants in grasslands with different topographies affect yak foraging preferences on the eastern Tibetan plateau

**DOI:** 10.3389/fpls.2024.1347576

**Published:** 2024-03-28

**Authors:** Jinlan Wang, Wenxia Cao, Hongmei Shi, Wen Li

**Affiliations:** ^1^ State Key Laboratory of Plateau Ecology and Agriculture, Qinghai University, Xining, China; ^2^ Grassland Ecosystem Key Laboratory of Ministry of Education, Sino-U.S. Research Centers for Sustainable Grassland and Livestock Management, College of Grassland Science, Gansu Agricultural University, Lanzhou, China; ^3^ Key Laboratory of Development of Forage Germplasm in the Qinghai-Tibetan Plateau of Qinghai Province, Qinghai Academy of Animal Science and Veterinary Medicine of Qinghai University, Xining, China; ^4^ Animal Husbandry Station, Gannan Tibetan Autonomous Prefecture, Hezuo, China

**Keywords:** Tibetan plateau, yak, forage nutritional quality, foraging preference, diet selection

## Abstract

Diet selection, a core problem of foraging behavior, is a nutritional adaptation strategy formed in the long-term natural selection process by grazing herbivores and is significant for the sustainable management of grassland. Studies have mainly focused on the impacts of the individual and whole community spatial characteristics and herbivore body status on herbivore foraging behavior; thus, the response and mechanism of forage plants in different terrains to the diet selection of grazing herbivores remains unclear. Therefore, in this study, forage plants (gramineae, cyperaceae, legume, forbs, edible shrubs, and community) in different topographies (terrace, riparian zones, shady slope, half shady slope, half sunny slope, sunny slope) on the eastern Tibetan plateau were selected to study changes in nutrient and mineral content characteristics of forage plants, as well as the difference in feeding bias of yaks for forage plants in different terrains by using an indoor cafeteria trial. A structural equation model was used to illustrate the impact of the forage plants in different terrains on the feeding bias of yak. The multi-criterion decision model TOPSIS showed that the nutritional value of gramineae was highest for the shaded slope, and that of cyperaceae and leguminosae was the highest for the terraces. The nutrient value of forbs and the whole community was highest for the sunny slope. Dry matter intake by yaks of leguminosae, forbs, and the whole plant community was significantly higher for terraces than for grasslands with other topographies, and all were significantly lower in riparian zones. Yak forage preference of leguminosae, forbs, and the whole community was the highest for the terrace and the lowest for the riparian zones. Structural equation modeling showed that for functional groups, the interactions between topography and functional groups were the drivers influencing yak forage preferences. Our study highlights the propensity of yaks to forage for plants in areas with different topographies. These results have provided a scientific basis for understanding the relationship between herbivores and plants in grasslands and for formulating scientific grazing management strategies, which are of considerable importance for sustainable grassland livestock husbandry.

## 1 Introduction

Herbivores are important determinants of ecosystem stability and plant communities. The foraging behavior of grazing herbivores is the most direct factor affecting plant resources and distribution pattern because of the voluntary feed intake and diet composition selected from the available pasture sward, and the quantity and the nutrient content of forages available (Dumont et al., 2007; Egelkraut et al., 2020). Diet selection, a core problem of foraging behavior, is a nutritional adaptation strategy formed in the long-term natural selection process by grazing herbivores. This directly affects the nutritional status of grazing herbivores from the environment and determines their dietary composition (Mancilla-Leytón et al., 2021). Diet selection has been described as the search for and selection of high-quality plant resources by examining the grassland topography, composition and structure of vegetation, as well as the feeding possibility and quality of herbage (Zhang et al., 2022; Fan et al., 2023), altering the plant competition by favoring one species over another, and affecting the structure and function of grassland plant communities (Wan et al., 2015; Oñatibia et al., 2023). Therefore, studying herbivore diet selection in grassland ecosystems to examine the mechanisms of grazing herbivore diet selection strategy to analyze the relationship between grazing herbivores and plants and the development of grassland grazing management techniques is essential.

The foraging behavior of grazing herbivores in natural grassland is highly complex and controlled by many factors, such as the plant heterogeneity induced by the change in grassland microenvironment (Huang et al., 2018), the plants of the same taxa exhibiting seasonal and partial differences in nutrient dynamics (Papachristou et al., 2005) and the physiological state and hunger levels of herbivores (Wang, 2010). Therefore, owing to the complexity of plant community distribution and the autogenous state of herbivores, diet selection is highly variable and uncertain (Liu et al., 2015). Many studies have investigated the foraging behavior of herbivores, mainly focusing on the spatial distribution of grassland plants (Wang et al., 2010; Huang et al., 2018), plant diversity (Feng et al., 2016), heterogeneous patch characteristics (Huang et al., 2012, 2018) and the influence of the degree of hunger of herbivores (Wang, 2010) on foraging behavior. These studies have mainly considered the effects of individual and whole community spatial characteristics and herbivore body status on herbivore foraging behavior. However, minimal information is available on the effect of different topographic grasslands. Thus, additional research is necessary to clarify the plants distributed in grasslands with different topographies that affect herbivore foraging behavior or the nature of the influencing mechanisms.

Yaks are endemic and most widely distributed on the Qinghai-Tibet Plateau. The foraging behavior of these poliphagous animals with selecting some species among various species influences the plant spatial distribution pattern greatly (Wang, 2022). Based on the aforementioned research, forage plants, namely, gramineae, cyperaceae, leguminosae, forbs, edible shrubs and the whole community in grasslands with different topographies, namely, terrace, riparian zones, shady slopes, half shady slopes, half sunny slopes and sunny slopes of the Tibetan plateau were selected to research the variation in the nutritional and mineral content of forage plants, as well as differences in yak forage preferences for forage plants in grasslands with different topographies by using an indoor cafeteria trial. These results provide novel insights into optimizing the yak grazing management system, improving grazing management level, the effective and stable utilization of grassland vegetation, preventing grassland ecological imbalance and maintaining grassland sustainable development.

## 2 Materials and methods

### 2.1 Study area

The experimental site is located in Zhaxixiulong township (N 37°11’, E 102°29’) in Tianzhu Tibetan Autonomous County, Gansu province, in the eastern part of the Qilian Mountains, with an average altitude of 3000 m. Between 1951 and 2018, the average annual temperature was 0.16°C and the average annual rainfall was 416.9 mm (Wang et al., 2021). The study area has a typical continental plateau climate, cold and humid, with thin air, strong solar radiation, water and heat at the same time. There is no absolute frost-free period, only cold and hot seasons, and the plant growth period is approximately 120 d. The main soil type is alpine chernozem. In the study area, the alpine shrub grassland was distributed in a band on the shaded slope, and the dominant shrubs were *Rhododendron thymifolium* and *R. capitatum*. The main herbaceous plants were *Polygonum viviparum* and *Equisetum arvense* (Wang et al., 2022). The shrub cover was approximately 50-80%, and the herb cover was approximately 60-85%. Other slopes in the study area were dominated by alpine meadows, with the dominant species being *Elymus nutans, Poa crymophila*, and *Kobresia humilis*, with herb cover of approximately 80% (Li et al., 2018).

### 2.2 Experimental design and samples

In mid-April 2019, the grassland in the study area was divided into terrace (0°), riparian zones (0°), half shady slope (0-45°, 315-360°), shady slope (45-90°, 270-315°), half sunny slope (90-135°, 225-270°) and sunny slope (135-225°). This was conducted according to the classification criteria of topography by Zha et al. (2010), combined with digital elevation model data and field investigation of grassland vegetation and soils. The elevation of the study area ranged from 2761 m to 4398 m. To examine the grassland vegetation of the study area in detail, this study set a 5 km wide transect every 300 m along the elevation, comprising a total of five transects. The terrace, riparian zones, shady slope, half shady slope, half sunny slope and sunny slope grassland were selected for each transect. The area of each grassland topography was at least 0.1 hm^2^. The grassland topography for each transect was sampled four times for each and arranged randomly. The distance between each two adjacent grassland topographies on each transect was at least 500 m. There were 24 plots per transect, comprising six with four replicates for each, with a total of 120 plots (5 plots ×24 plots/transect = 120 plots). The vegetation and soil of the grasslands in July 2019 are listed in **Supplementary Table S1**.

In mid-July 2019, three yak adult cows of similar weight, approximately 180 ± 5 kg, were selected as test animals to conduct a simulated feeding experiment using an indoor cafeteria trial according to the methods of Wang (2010). Before the formal feeding experiment, the yaks were trained and familiarized with the transition for two weeks. They were fed fresh forage samples and a basic concentrate diet during the transitional period. This protocol was used to allow them to adapt to the formal test feeding mode and forage cutting and reach a stable and healthy nutritional state before the formal experiment. The formal feeding experiment was conducted in early August 2019. gramineae, cyperaceae, leguminosae, forbs, edible shrubs comprising newly-grown branches and leaves, and the whole community in grasslands with different topographies were collected and stored in cold cellars the day before the formal feeding experiment. To eliminate the influence of plant diversity on yak foraging preferences, we consistently maintained the species and quantity of forage plants in each topographical grassland (**Supplementary Table S2**). The forage plants were cut to approximately 10 cm before feeding, and the feeding experiment was conducted for 5 h after a full meal. During the feeding experiment, the yaks were placed in a separate enclosure, and forage plants from grasslands with different topographies were placed in the trough. Each trough was filled with an equal number of forage plants, and yaks were allowed to consume them freely for 1 h. Forage plant intake was calculated as the difference in the weight of forage plants before and after the feeding experiment. The forage preference index was calculated as the percentage of forage plant intake from each functional group, to the total forage plant intake.

### 2.3 Sample analyses

For the forage plants, samples of approximately 400 g from each functional group were collected and transported to the laboratory. They were immediately deoxidized at 105°C for 30 min before being oven-dried at 70°C to constant weight. They were then milled through a 1-mm sieve for analysis of the content of crude protein (CP), crude fat (EE), crude ash (Ash), crude fiber (CF), acid detergent fiber (ADF), neutral detergent fiber (DNF), and the mineral elements phosphorus (P), potassium (K), sodium (Na), calcium (Ca), magnesium (Mg), iron (Fe), manganese (Mn), copper (Cu), zinc (Zn) and cobalt (Co). We measured CP using a Kjeldahl analyzer (Kjeltec 2300, Hoganas, Sweden), EE was analyzed by conducting 550°C muffle furnace incineration (SX2-2.5-10TP), NDF and ADF were analyzed using an Ankom 2000 fiber analyzer (Ankom Technology, Fairport, NY, USA) according to the methods of Bao (2000). The nitrogen free extract (NFE) and relative feeding values (RFV) were then calculated. The mineral elements were determined using an AFG atomic absorption spectrophotometer (TAS-990). The specific formula is as follows (Linn and Martin, 1991):

NFE = 100%- (EE%+CF%+CP%+Ash%)

RFV = (DMI(%) × DDM(%))/1.29

DMI (%) = 120/(NDF(%))

DDM (%) = 88.9- ADF(%)

where DMI is dry matter intake and DDM is digestible dry matter.

### 2.4 Statistical analysis

Microsoft Excel 2019 was used to organize the data, and SPSS software (SPSS 19.0, Chicago, IL, USA) was used to test the normality (Kolmogorov–Smirnov) and homogeneity of variance of forage plant nutrition and mineral elements. Multiple comparisons (LSD) using one-way ANOVA in SPSS software were used to analyze the significance of the same index among grasslands with different topographies (*P* < 0.05). Based on the content of various nutrients of forage plants in grasslands with different topographies, a multi-criterion decision model-TOPSIS (Technique for order preference by similarity to an ideal) with R 4.0.2 (R Development Core Team) plyr data package was used to comprehensively evaluate the nutritional status of forage plants in functional groups and communities of grassland with different topographies. To investigate the relative contributions of nutrient and mineral element contents of forage plants in grasslands with different topographies to yak foraging preference, we used the randomForest package in R 4.0.2 to rank them. The factors that contributed significantly to yak forage preference change were labeled. The variance decomposition of the MuMIn package (R 4.0.2) was used to research the relative contribution rates of the main plant nutrition and mineral element factors to yak forage preference. Using the R 4.0.2 (R Development Core Team) piecewise structural equation model (SEM) package, a piecewise structural equation model was constructed to examine how topography, plant functional groups, and their interactions affected plant nutrition and mineral content through different paths, and then affected yak foraging preference. Origin software (version 8.0) was used for drawing.

## 3 Results

### 3.1 Nutritional value of forage plants in grasslands with different topographies

**Figure 1:**
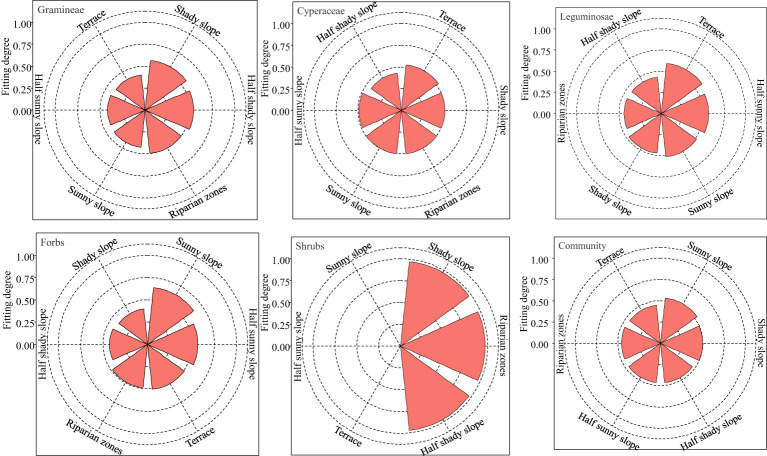
Detailed evaluation of forage plant nutrition on different grassland topographies on the Tibetan plateau.

The multi-criterion decision model TOPSIS was used to comprehensively evaluate the nutritional value of gramineae, cyperaceae, leguminosae, forbs, edible shrubs and the whole community in grasslands with different topographies (**Figure 1**). The degree of nutrition fitting of gramineae was the highest in the shady slope with 0.57. That of cyperaceae and leguminosae was the highest in the terrace, with 0.53 and 0.60, respectively. Forbs and the whole community were the highest in the sunny slope, with 0.64, and 0.50, respectively. That of edible shrubs was the highest for the shady slope, riparian zones, and half shady slope, with 0.97, 0.97, and 0.97, respectively. Therefore, the nutritional value of gramineae, cyperaceae, leguminosae and forbs was highest on the shady slope, terrace, terrace, and sunny slope, respectively. The nutritional quality of the whole community was highest on the sunny slope.

### 3.2 Yak foraging preferences between forage plants from different grassland topographies

#### 3.2.1 Dry matter intake by yak of forage plants from different grassland topographies

**Figure 2:**
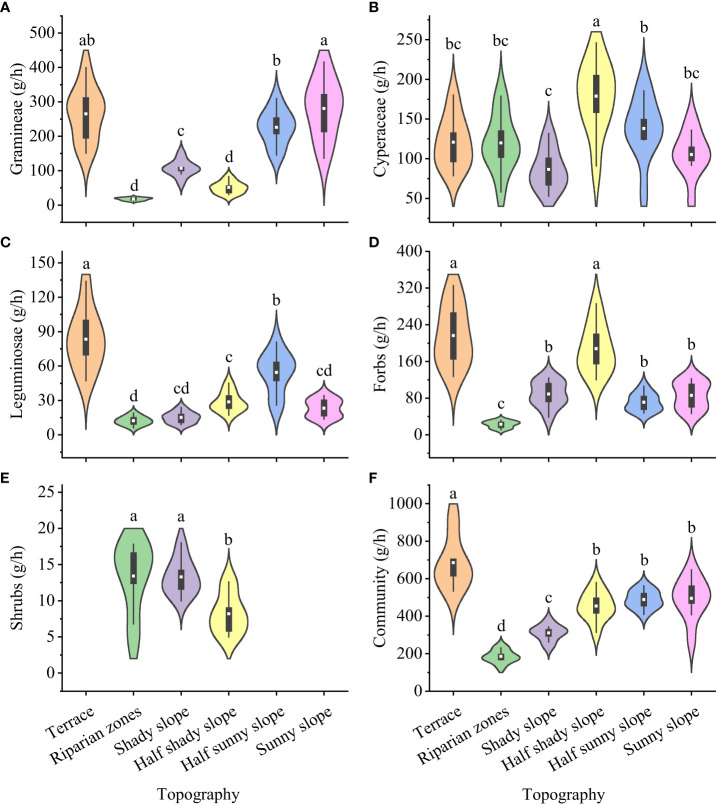
Dry matter intake of forage plants by yak from grasslands with different topographies on the Tibetan plateau. **(A)** Gramineae; **(B)** Cyperaceae; **(C)** Leguminosae; **(D)** Forbs; **(E)** Shrubs; **(F)** Community. Lowercase letters represent significant differences in the different terrains (P<0.05).

The dry matter intake of yaks differed for gramineae, cyperaceae, leguminosae, forbs, edible shrubs, and the whole community in grasslands with different topographies (**Figure 2**): gramineae, significantly highest for the sunny slope (280.73 g/h) and significantly lowest for the riparian zones and half shady slope (17.38 g/h and 50.91 g/h, respectively); cyperaceae, significantly highest for the half shady slope (178.78 g/h) and significantly lowest for the shady slope (86.47 g/h); leguminosae, significantly highest for the terrace (83.40 g/h) and significantly lowest for the riparian zones (12.22 g/h); forbs, significantly highest for the terrace and half shady slope (216.42 g/h and 187.80 g/h, respectively) and significantly lowest for the riparian zones (22.78 g/h); edible shrubs, significantly highest for the riparian zones and shady slope (13.40 g/h and 13.29 g/h, respectively) and significantly lowest for the half shady slope (8.20 g/h); and the whole community, significantly highest for the terrace (685.40 g/h) and significantly lowest for the riparian zones (185.69 g/h).

#### 3.2.2 Forage preference of yak to different topography grassland forage plants

**Figure 3:**
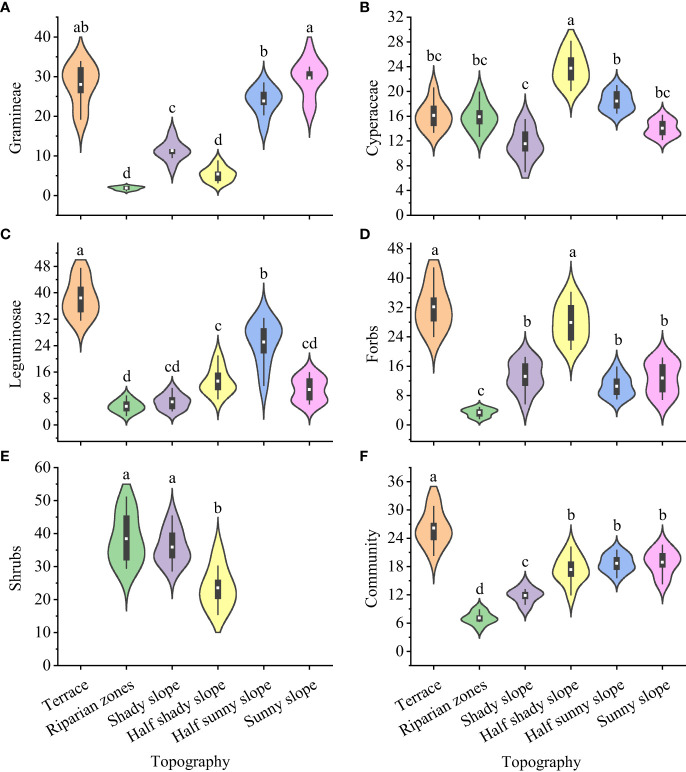
Forage preference index for forage grass for yak from different topographies on the Tibetan plateau. **(A)** Gramineae; **(B)** Cyperaceae; **(C)** Leguminosae; **(D)** Forbs; **(E)** Shrubs; **(F)** Community. Lowercase letters represent significant differences in the different terrains (P<0.05).

The forage preference index of yaks differed significantly for gramineae, cyperaceae, leguminosae, forbs, edible shrubs, and the whole community in grasslands with different topographies (**Figure 3**): gramineae, highest for the sunny slope (29.66) and lowest for the riparian zones and half shady slope (1.84 and 5.38, respectively); cyperaceae, highest for the half shady slope (23.75) and lowest for the shady slope (11.54); leguminosae, highest for the terrace (38.42) and lowest for the riparian zone (5.63); forbs, highest for the terrace and half shady slopes (32.17 and 27.92, respectively) and lowest for the riparian zones (3.39); edible shrubs, highest for the terrace and shady slopes (38.41 and 35.87, respectively) and lowest for the half shady slope (23.50); and the entire community, highest for the terrace (6.16) and significant and the lowest for the riparian zone (7.09).

#### 3.2.3 Forage preference mechanisms of yak to grassland plants from areas with different topographies

**Figure 4:**
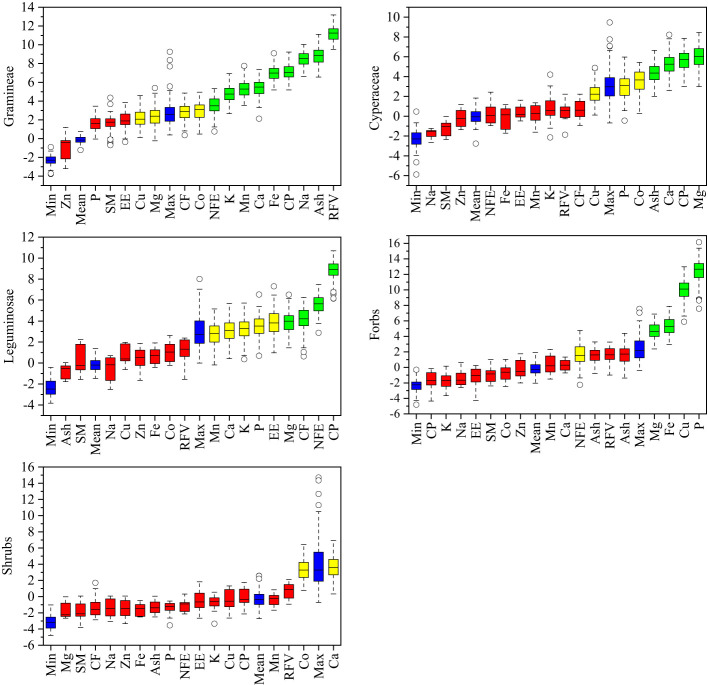
Importance of variable factors in yak forage preference. CP, crude protein; EE, crude fat; Ash, crude ash; CF, crude fiber; NFE, nitrogen free extract; RFV, relative feeding values; P, phosphorus; K, potassium; Na, sodium; Ca, calcium; Mg, magnesium; Fe, iron; Mn, manganese; Cu, copper; Zn, zinc; Co, cobalt.

To investigate the relative contribution of plant nutrition and mineral element factors to the forage preference of yaks, the importance of contributing factors to the forage preference of gramineae, cyperaceae, leguminosae, forbs, edible shrubs, and the whole community was ranked based on the random forest principle. As shown in **Figure 4**, the factors that contributed the most to the forage preference of gramineous plants were RFV, Ash, Na, CP, Fe, Ca, Mn, K, NDF, Co, and CF. Mg, CP, Ca, Ash, Co, and P contributed the most to the forage preference for cyperaceae. The factors that contributed the most to the forage preference for legumes were CP, NFE, CF, Mg, EE, P, K, Ca, and Mn. The factors that contributed the most to forage preference for forbs were P, Cu, Fe, and Mg. The most important factor contributing to the forage preference for edible shrubs was Ca.

**Figure 5:**
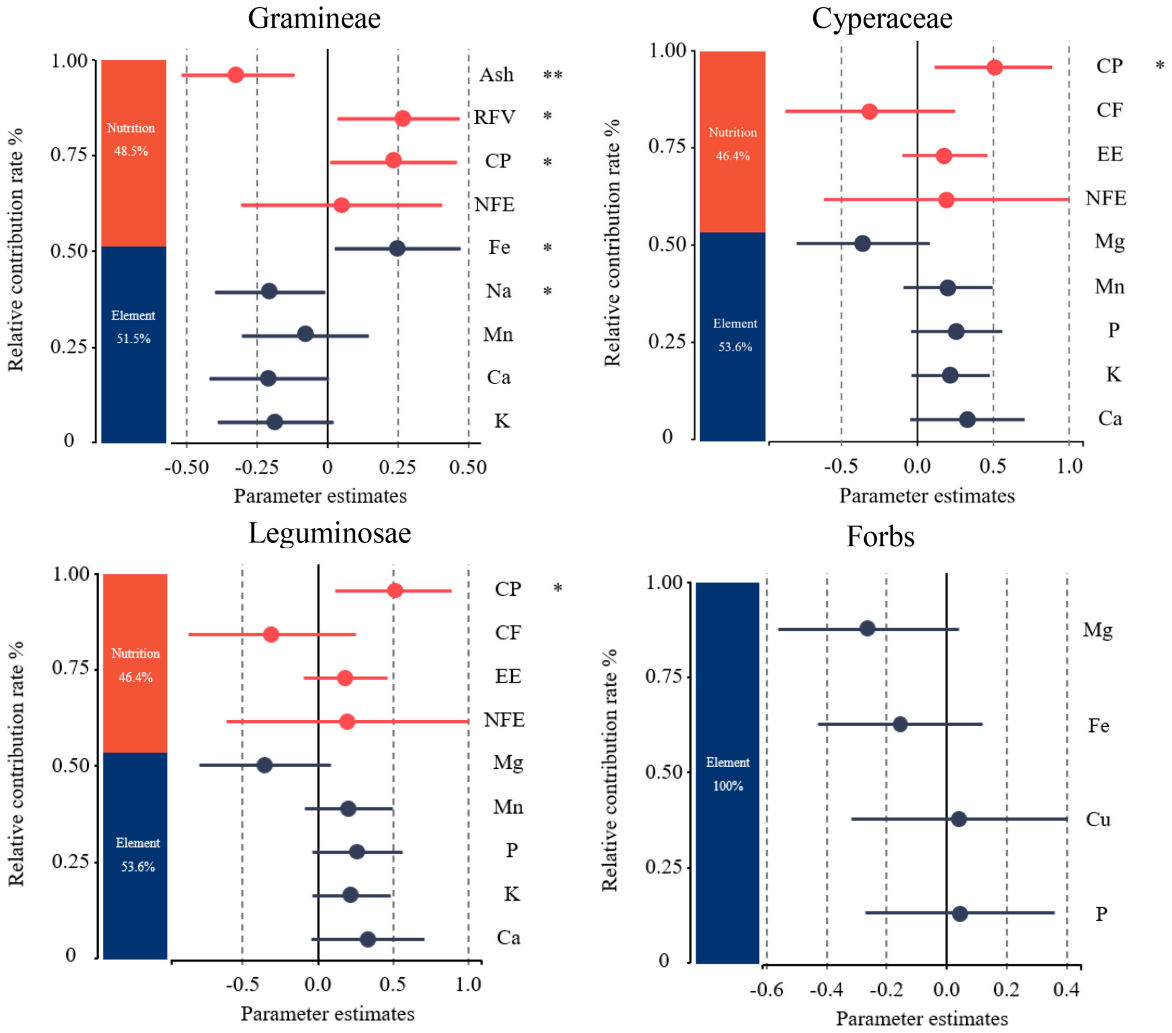
Relative contribution rate changes of forage plant factors to yak forage preferences on the Tibetan plateau. CP, crude protein; EE, crude fat; Ash, crude ash; CF, crude fiber; NFE, nitrogen free extract; RFV, relative feeding values; P, phosphorus; K, potassium; Na, sodium; Ca, calcium; Mg, magnesium; Fe, iron; Mn, manganese; Cu, copper. *P < 0.05, **P < 0.01.

The factors with the greatest contribution to yak forage preference from **Figure 4** were selected for further variance decomposition (**Figure 5**). Plant nutrition contributed 48.5% to the gramineae forage preference of yaks, and mineral elements contributed 51.5%, among which the contents of CP, RFV, and Fe had a significantly positive effect, Na and Ash had a significantly negative effect. As for the cyperaceae forage plants, plant nutrition contributed 49% to yak forage preference, and mineral elements contributed 51%, among which the P content showed a significantly positive correlation, meanwhile, the CF content showed a significantly negative correlation. As for leguminosae forage plants, plant nutrition contributed 46.4% to yak forage preference with CP content had a significantly positive effect, and mineral elements contributing 53.6%. Mineral elements contributed 100% to forbs forage preference of yak.

**Figure 6:**
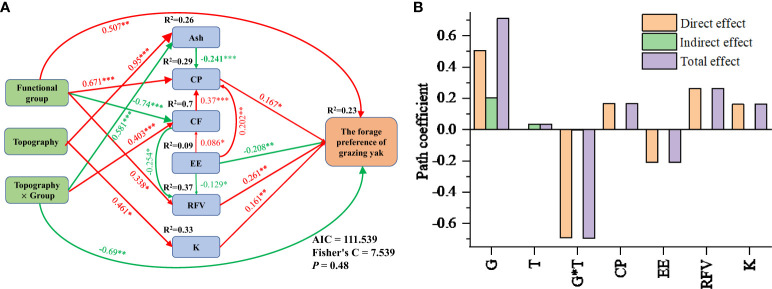
Effects of forage plant nutrition and mineral element content variables on yak forage preference calculated using the structural equation model **(A)**. Direct, indirect and total effects of forage plant nutrition and mineral element content variables on yak forage preference **(B)**. Ash, crude ash; CP, crude protein; CF, crude fiber; EE, crude fat; RFV, relative feeding values; K, potassium. Red solid and green solid arrows represent significantly positive or negative effects at the 0.05 level, respectively. Significant standard path coefficients are shown on arrows and proportionate to the arrow thickness. *P < 0.05, **P < 0.01, ***P < 0.001.

Factors that had a significant impact on the variance decomposition of yak forage preference for each functional group were selected (**Figure 6**). A segmental structural variance model was further established to explore how topography, plant functional groups, and their interactions affected plant nutrition and mineral element content through different paths, thus affecting yak forage preference (**Figure 6A**). The model was well fitted, and the *P* value, Fisher’s C value and Akaike information criterion (AIC) values of the model were 0.48 (>0.05), 7.539, and 111.539, respectively. The results of the model showed that the functional groups of forage plants had a direct positive effect on yak forage preference, with a path coefficient of 0.507. Functional groups also had indirect effects on yak forage preference by affecting forage plant CP, RFV, and CF. Meanwhile, topography indirectly affected yak forage preferences by influencing the contents of Ash and K elements in the forage plants. The interaction between topography and functional groups had a direct negative effect on yak forage preference, with a path coefficient of −0.69. The interaction between terrain and functional groups also indirectly affected yak forage preference by influencing the Ash and CF content of forage plants. CP, RFV, and K had significant positive effects on yak forage preference, with path coefficients of 0.167, 0.261 and 161, respectively. Meanwhile, EE had a significant negative effect on yak forage preference, with a path coefficient of −0.261.

Based on the standard path coefficients in the segmented structural equation, we calculated the direct, indirect, and total effects on yak forage preferences (**Figure 6B**). The total effects of functional group and interaction between topography and functional groups on the yak forage preference were higher, at 0.71 and −0.69, respectively. Therefore, functional groups and the interaction between topography and functional groups were the main factors influencing yak forage preference.

## 4 Discussion

Plant nutrition reflects the ability of plants to absorb and store nutrients from the soil in a specific habitat (Singh and Dutta, 2021). Therefore, it plays a key role in the main energy sources and nutrition for herbivores (Wang and Hou, 2021). The nutritional quality of forage plants is usually determined by the content of conventional chemical components, especially the crude protein and fiber (Alibayev et al., 2021). The topography induced the receipt and redistribution of light, heat, and water, affecting the plant nutrition (Liu et al., 2019). Our research demonstrated that the crude protein contents of gramineae, cyperaceae, leguminosae, and forbs from the sunny slope were higher than those in grasslands with other topographies (**Supplementary Figure S3**). The crude protein content of forage plants is correlated with the environmental temperature (Vanova and Marinovad, 2014). Sunny slope has relatively long-term solar radiation and habitats with relatively high temperatures would result in the growth rate of the accumulated dry matter of plants lagging behind that of nitrogen accumulation (Jiang et al., 2022b). Therefore, the crude protein content of forage plants is higher. Fiber content is also a key factor affecting the quality and feeding value of forage plants. Therefore, forage plants with low fiber content have a high level of palatability (Yang et al., 2021). In this study, the crude fiber content of gramineae, cyperaceae, leguminosae and forbs for sunny slopes was higher than that of grasslands with other topographies. This may be attributed, to some extent, to the longer photosynthetic time during the daytime on the former than the latter. This accumulated more non-structural carbohydrates, increasing the contents of insoluble secondary wall structural carbohydrates and lignin, further resulting in a higher plant fiber content (Jin et al., 2017). Mineral elements are indispensable nutrients for the growth and development of forage plants and play a key role in cell division (Brengi et al., 2022), protein synthesis (Johnson et al., 2022), metabolism (Sadeghi et al., 2021), and redox processes (Tavanti et al., 2020). The contents of Ca, Mg, Mn and Co in gramineae, cyperaceae, leguminosae and forbs on shady slopes and half shady slopes were higher than those in other topographical grasslands in the current study (**Supplementary Figure S4**). This may be due to the high SOM content of soil organic matter for the shady and half shady slopes (**Supplementary Table S1**). Soil organic matter content will positively promote the absorption of mineral elements by roots, increasing the plant mineral element content (Daryabeigi Zand and Mühling, 2022). Abundant soil carbon and nitrogen sources can promote the dissolution of mineral elements and form soluble complexes for the absorption of forage plants, increasing forage plant content (Jiang et al., 2022a). Soluble organic matter increased the content of mineral elements in the soil and improved the transport capacity of the root system for mineral elements, resulting in a higher content of vegetation elements in the shady and half shady slopes.

The feeding behavior of herbivores is an effective strategy for adapting to the environment and fulfilling their needs (Mellado et al., 2011). Nutrients taken up by herbivores through foraging can be used for production if they satisfy physiological maintenance (Singh and Dutta, 2021). Forage preference indicates that herbivores show a degree of preference for certain plants when they fulfill their nutritional needs for growth and reproduction to the maximum extent (Cougnon et al., 2018). Diet selection in domestic animals is regulated by factors such as post-ingestive feedback from the digest, the distribution pattern of grassland plant resources and plant nutrient content (Pain et al., 2015). In our research, the factors of domestic animals were the same when using an indoor cafeteria trial, and diet selection in domestic animals was mainly affected by plant factors (mainly nutrient content and mineral elements). Our results showed that the dry matter intake and forage preference of yaks in terraces were higher than those in grasslands of other topographies, and the multi-criterion decision model TOPSIS analysis revealed that the nutritional quality of the whole community was lowest in the terraces. This result conflicts with a result in the literature: herbivores preferentially consume forage plants with high nutritional value (Zhang et al., 2022). An explanation for this difference might be as follows: owing to the higher content of Zn and Cu (**Supplementary Table S4**) on the terrace to fulfill the physiological maintenance and reproductive needs of yaks during the time or season, which further confirmed livestock nutritional wisdom, livestock would show a desire for the nutrient/element and consume food containing large amounts of the nutrient/element when a certain nutrient/element is insufficient in their bodies (Wang and Wand, 2007). Environment heterogeneity induced the lowest content of P, K, Na, Ca, Mg, Fe, Mn, Co, and nutrition for the terrace, and compared with other topographies grassland, herbivores were maximally consumed to compensate for lower sward availability (Zhang et al., 2022). Additionally, grazer diet quality decreases with increasing body size (Codron et al., 2007), thus, herbivores must constantly make trade-offs against some factors to survive, grow and reproduce, and sometimes, yaks with large body sizes selected forage plants with lower nutritional requirements. Therefore, yaks preferentially consumed forage plants on the terraces. Additionally, yak forage preference of leguminosae and forbs was highest in the terrace, and the SEM analysis showed that functional groups was the main factors influencing yak forage preference. An explanation for the result might be the characteristics of functional groups (Bitomský et al., 2023). Plant functional groups have similar responses to environmental factors based on plant morphology, physiology and life history (Zhang et al., 2018), and the characteristics of functional groups are mainly affected by genetic and environmental factors. For the terrace, leguminosae had higher nutrition quality and higher P content, which would fulfill the requirements of yaks, and forbs had the lowest nutrition quality and low element content (except Zn), resulting in the herbivores exhibiting maximal consumption to compensate for lower sward availability (Zhang et al., 2022).

## 5 Conclusion

On the Tibetan plateau, the nutritional value of gramineae was highest for the shaded slope, that of cyperaceae and leguminosae was highest for the terrace, and that of forbs and the whole community was highest for the sunny slope. Forage plants in grasslands with different topographies significantly affected the dry matter intake and yak forage preferences. The dry matter intake and yak forage preference of leguminosae, forbs, and the whole community were significantly higher for terraces than for grasslands with other topographies. The segmental structural equation showed that functional group and the interaction between topography and functional groups were the main influencing factors of yak forage preference. Our result provides data support for yak grazing management systems and effective and sustainable utilization of grassland vegetation.

## Data availability statement

The original contributions presented in the study are included in the article/**Supplementary Material**. Further inquiries can be directed to the corresponding author.

## Author contributions

JW: Data curation, Investigation, Writing – original draft. WC: Conceptualization, Data curation, Funding acquisition, Methodology, Resources, Writing – review & editing. HS: Conceptualization, Formal analysis, Methodology, Writing – review & editing. WL: Conceptualization, Formal analysis, Methodology, Writing – review & editing.
