# Supplementary material for: Forage plants in grasslands with different topographies affect yak foraging preferences on the eastern Tibetan plateau
Source: Front Plant Sci. 2024 Mar 28;15:1347576. doi: 10.3389/fpls.2024.1347576 (PMC11010683; doi:10.3389/fpls.2024.1347576)
Supplement: Supplementary file 1 [file Table_1.docx]

**Forage plants in grasslands with different** **topographies affect yak foraging preferences** **o****n the** **eastern Tibetan plateau**

Jinlan Wang ^1,2, 3^, Wenxia Cao ^2^, Hongmei Shi ^4^, Wen Li ^3,2*^

^1^ State Key Laboratory of Plateau Ecology and Agriculture, Qinghai University, Xining 810016, China

^2^ Grassland Ecosystem Key Laboratory of Ministry of Education, Sino-U.S. Research Centers for Sustainable Grassland and Livestock Management, College of Grassland Science, Gansu Agricultural University, Lanzhou 730070, China

^3^ Key Laboratory of Development of Forage Germplasm in the Qinghai-Tibetan Plateau of Qinghai Province, Qinghai Academy of Animal Science and Veterinary Medicine of Qinghai University, Xining 810016, China

^4^ Animal Husbandry station, Gannan Tibetan autonomous Prefecture, Hezuo, 747000, China

∗ Corresponding author. Tel.: +86 931 7632829. Fax.: +86 931 7631227

E-mail address: lw@qhu.edu.cn

Table S1 Grassland vegetation and soil characteristics of in grasslands with different topographies of the Tibetan plateau

|  | Terrace | Riparian zones | Shady slope | Half shady slope | Half sunny slope | Sunny slope |
| --- | --- | --- | --- | --- | --- | --- |
| Dominant species | *Elymus nutans*  *Poa pratensis*  *Stipa aliena*  *Kobresia humilis* | *Carex orbicularis*  *P. pratensis* | *C. atrofusca*  *P. pratensis*  *E. nutans*  *C. orbicularis* | *E. nutans*  *P. pratensis* | *S. aliena*  *Leymus secalinus*  *K. capillifolia* | *S. aliena*  *P. pratensis*  *L. secalinus* |
| Herbaceous height (cm) | 32.35±1.17a | 22.65±0.82c | 19.4±0.66d | 19.26±0.7d | 23.6±0.85c | 26.1±0.94b |
| Herbaceous coverage (%) | 95.00±1.73a | 82.00±0.58c | 69.00±1.73e | 93.00±1.15a | 87.00±1.73b | 76.00±1.15d |
| BD in 0-20 cm layer (g/m^3^) | 0.92±0.04bc | 1.11±0.04a | 0.74±0.03d | 0.81±0.03cd | 0.93±0.04bc | 0.96±0.04b |
| SM in 0-20 cm layer (%) | 34.51±1.24c | 28.52±1.03d | 43.58±1.57a | 38.74±1.39b | 26.7±0.96de | 24.3±0.88e |
| SOM in 0-20 cm layer (g/kg) | 34.51±1.87b | 28.54±1.82c | 43.57±3.05a | 38.73±1.19b | 26.73±1.12d | 24.32±1.2d |
| TN in 0-20 cm layer (g/kg) | 6.6±0.19b | 6.29±0.21bc | 7.69±0.22a | 6.82±0.19b | 5.6±0.15d | 6±0.13cd |

Note: Different lowercase letters represent significant differences in the different terrains (*P*<0.05).

Table S2 The species and species number of functional group during formal feeding experiment

|  | Gramineae | Cyperaceae | Leguminous | Forbs | Edible shrubs | Whole community |
| --- | --- | --- | --- | --- | --- | --- |
| Species | *Elymus nutans,*  *Poa pratensis, Koeleria cristata,*  *Leymus secalinus* | *Carex digyne,*  *C. aridula,*  *Kobresia humilis,*  *K. capillifolia* | *Astragalus monadelphus,*  *A. polycladus, Medicago ruthenia var. inschanica,*  *Gueldenstaedtia diversfolia* | *Taraxacum mongolicum,*  *Potentilla discolor,*  *Polygonum viviparum,*  *Plantago depressa* | *Salix oritrepha,*  *S. rehderiana,*  *S. sclerophylla,*  *Caragana jubata* | *Elymus nutans,*  *Poa pratensis, Koeleria cristata,*  *Leymus secalinus, Carex digyne,*  *C. aridula,*  *Kobresia humilis,*  *K. capillifolia,*  *Astragalus monadelphus,*  *A. polycladus, Medicago ruthenia var. inschanica,*  *Gueldenstaedtia diversfolia,*  *Salix oritrepha,*  *S. rehderiana,*  *S. sclerophylla,*  *Caragana jubata* |
| Species Number | 4 | 4 | 4 | 4 | 4 | 16 |

Table S3 Nutrient characteristics of forage plants in grasslands with different topographies of the Tibetan plateau

|  | Topography | Crude protein% | Crude fat % | Crude ash % | Crude fibre % | Acid detergent fiber **%** | Neutral detergent fiber **%** | Nitrogen Free Extract % | Relative feeding value |
| --- | --- | --- | --- | --- | --- | --- | --- | --- | --- |
| Gramineae | Terrace | 8.7b | 2.62ab | 7.56c | 28.8bc | 52.32bc | 47.99ab | 52.31bc | 50.83b |
|  | Riparian zones | 6.7c | 2.27abc | 10.18a | 29.4bc | 51.45bc | 41.27bc | 51.45bc | 65.54a |
|  | Shady slope | 7.2c | 2.06c | 9.3ab | 25.6d | 55.84a | 40.14c | 55.84a | 71.68a |
|  | Half shady slope | 7.6bc | 2.15bc | 8.4bc | 27.8cd | 54.05ab | 42.2bc | 54.05ab | 66.87a |
|  | Half sunny slope | 8.6b | 2.74a | 7.7c | 31.4b | 49.56c | 50.38a | 49.56c | 51.88b |
|  | Sunny slope | 9.92a | 2.71a | 7.45c | 36.9a | 43.02d | 52.89a | 43.04d | 46.01b |
| Cyperaceae | Terrace | 11.5a | 2.61ab | 7.49b | 26.7c | 51.7bc | 38.83bc | 51.7bc | 65.62bcd |
|  | Riparian zones | 9.6bc | 2.02c | 9.26a | 28.5bc | 50.62cd | 37.12c | 50.62cd | 74.51ab |
|  | Shady slope | 9.2c | 2.15bc | 9.52a | 24.3d | 54.83a | 35.11c | 54.83a | 77.14a |
|  | Half shady slope | 10.8ab | 2.37abc | 6.18c | 26.5c | 54.15ab | 36.35c | 54.15ab | 68.73abc |
|  | Half sunny slope | 11.9a | 2.53abc | 7.05bc | 30.5b | 48.02d | 47.14a | 48.02d | 56.26d |
|  | Sunny slope | 12a | 2.76a | 7.31bc | 33.6a | 44.33e | 44.42ab | 44.33e | 60.1cd |
| Legume | Terrace | 17.3a | 3.42a | 8.56b | 21.4d | 49.32c | 38.72a | 49.32c | 82.14bc |
|  | Riparian zones | 13.9b | 2.13c | 7.31c | 24.3c | 52.36ab | 35.41a | 52.36ab | 100.59a |
|  | Shady slope | 12.95b | 2.53bc | 10.86a | 20.1d | 53.56a | 37.2a | 53.56a | 92.63ab |
|  | Half shady slope | 13.6b | 2.46bc | 9.96a | 23.4c | 50.58bc | 36.74a | 50.58bc | 80.55bcd |
|  | Half sunny slope | 17.8a | 2.93ab | 10.14a | 26.4b | 42.73d | 37.17a | 42.73d | 74.27cd |
|  | Sunny slope | 16.8a | 3.3a | 8.47bc | 29.3a | 42.13d | 40.93a | 42.13d | 66.18d |
| Forbs | Terrace | 10.5ab | 2.74a | 10.77c | 17.9cd | 58.09b | 41.13a | 58.09b | 77.75bc |
|  | Riparian zones | 9.23b | 2.11b | 9.27d | 17.6cd | 61.42a | 38a | 61.79a | 93.29ab |
|  | Shady slope | 10.35ab | 2.93a | 8.43d | 16.2d | 62.09a | 39.33a | 62.06a | 94.79ab |
|  | Half shady slope | 10.4ab | 2.86a | 8.43d | 18.9c | 59.41ab | 40.52a | 59.41ab | 101.45a |
|  | Half sunny slope | 9.5b | 2.85a | 12.27b | 21.3b | 54.08c | 42.82a | 54.08c | 66.25c |
|  | Sunny slope | 10.9a | 3.1a | 13.65a | 24.5a | 47.85d | 38.75a | 47.85d | 81.84abc |
| Edible shrubs | Riparian zones | 12.7a | 3.4a | 6.5a | 24.8b | 52.6a | 41.1a | 55.10a | 85.26a |
|  | Shady slope | 13.5a | 3.2a | 6.8a | 27.9a | 48.6b | 40.3a | 50.90b | 87.89a |
|  | Half shady slope | 13.6a | 3.1a | 6.4a | 26.6a | 50.3b | 38.1a | 52.70b | 90.36a |

Note：Shrubs only appeared in riparian zones, shady slope and half shady slope grassland. Different lowercase letters represent significant differences in the different terrains (*P*<0.05).

Table S4 Elements contents of forage plants in grasslands with different topographies of the Tibetan plateau

|  | Topography | P  g/kg | K  g/kg | Na  g/kg | Ca  g/kg | Mg ug/g | Fe  g/kg | Mn  g/g | Co  ug/g | Zn  ug/g | Cu  ug/g |
| --- | --- | --- | --- | --- | --- | --- | --- | --- | --- | --- | --- |
| Gramineae | Terrace | 1.94b | 94.2b | 14.74b | 0.51d | 2.16b | 0.51cd | 116.62bcd | 295.6e | 2773.5bc | 456.3b |
|  | Riparian zones | 1.1d | 117.4a | 18.25a | 0.41e | 1.35c | 0.45d | 121.09bc | 334.2d | 2622.8c | 357.6d |
|  | Shady slope | 2.16a | 93.2b | 14.8b | 0.85a | 3.16a | 0.53bc | 142.5a | 390.2c | 3147.3a | 503.3a |
|  | Half shady slope | 1.96b | 110.9a | 16.96ab | 0.71b | 2.08b | 0.49cd | 127.87b | 543.7a | 2709.3bc | 483.6ab |
|  | Half sunny slope | 1.4c | 105.6ab | 16.32ab | 0.59c | 1.04d | 0.58b | 103.8d | 504.11b | 2654.3bc | 393.5cd |
|  | Sunny slope | 1.13d | 95.3b | 15.1b | 0.45de | 1.5c | 0.65a | 108.1cd | 412.3c | 2866.2b | 412.6c |
| Cyperaceae | Terrace | 1.3c | 98.3b | 15.32a | 1.75cd | 2.48c | 0.51bc | 115.16bc | 292.7d | 2905.2b | 432.3b |
|  | Riparian zones | 0.98d | 134.2a | 17.51a | 1.51e | 1.64d | 0.47c | 109.3c | 279.6d | 2423.6c | 374.2c |
|  | Shady slope | 1.92a | 113.6b | 15.59a | 2.65a | 3.89a | 0.5bc | 134.2a | 380.6ab | 3352.3a | 508.7a |
|  | Half shady slope | 1.8a | 143.85a | 14.72a | 2.09b | 3.24b | 0.49c | 123.5ab | 332.11c | 3113.7ab | 486.3a |
|  | Half sunny slope | 1.51b | 130.6a | 15.43a | 1.95bc | 2.42c | 0.56ab | 105.6c | 346.9bc | 2986.33b | 410bc |
|  | Sunny slope | 1.02d | 108.4b | 14.93a | 1.7de | 1.72d | 0.57a | 115.4bc | 406.3a | 2865.3b | 432.2b |
| Leguminous | Terrace | 2.14a | 127.89ab | 15.75a | 4.64c | 4.49c | 0.53b | 137.2bc | 339.6bc | 3025.9b | 513.4a |
|  | Riparian zones | 1.96b | 101.56c | 18.53a | 4.13d | 3.67d | 0.51bc | 124.3c | 305.7c | 2866.7b | 412.6b |
|  | Shady slope | 1.5c | 120.3b | 17.21a | 5.7a | 6.57a | 0.47c | 124.2c | 321.5c | 2986.9b | 549.6a |
|  | Half shady slope | 2.1ab | 138.9a | 16.43a | 5.2b | 5.18b | 0.49bc | 135.2bc | 375.2b | 2895.2b | 508.9a |
|  | Half sunny slope | 1.11e | 142.3a | 17.79a | 4.82bc | 2.35f | 0.6a | 144.3b | 413.2a | 3105.6ab | 422.3b |
|  | Sunny slope | 1.31d | 121.2b | 17.95a | 3.8d | 2.95e | 0.64a | 160.3a | 421.6a | 3343.3a | 441.3b |
| Forbs | Terrace | 1.84c | 130.94b | 17.03a | 3.95a | 3.35c | 0.55c | 124.8bc | 325.6bc | 2707.5b | 534.3a |
|  | Riparian zones | 1.41d | 127.9b | 18.25a | 3.98a | 3.12cd | 0.53c | 128.9abc | 298.4c | 2461.2b | 404.1b |
|  | Shady slope | 2.73a | 134.5b | 17.15a | 4.26a | 4.28a | 0.54c | 140.2a | 334.8b | 2467.7b | 396.4b |
|  | Half shady slope | 2.15b | 136.59b | 17.65a | 3.86ab | 3.76b | 0.52c | 118.4c | 342.3b | 2568.9b | 391.4b |
|  | Half sunny slope | 1.69c | 138.6b | 15.81a | 3.49bc | 2.76d | 0.61b | 116.3c | 404.43a | 2978.4a | 376.7b |
|  | Sunny slope | 1.35d | 160.52a | 16.32a | 3.4c | 2.93d | 0.68a | 136.68ab | 412a | 3120.9a | 401.2b |
| Edible shrubs | Riparian zones | 1.97b | 124.6a | 17.89a | 3.42ab | 3.95a | 0.57b | 153.2a | 385.6a | 2841.3a | 476.3b |
|  | Shady slope | 2.2a | 131.4a | 17.22a | 3.25b | 4.02a | 0.62a | 150.3ab | 395.8a | 2895.6a | 524.3a |
|  | Half shady slope | 2.05b | 127.5a | 18.21a | 3.65a | 3.52b | 0.59ab | 142.3b | 412.3a | 2854.3a | 489.3b |

Note：Shrubs only appeared in riparian zones, shady slope and half shady slope grassland. Different lowercase letters represent significant differences in the different terrains (*P*<0.05).
